# Exploring the Mechanism of Ferroptosis Induction by Sappanone A in Cancer: Insights into the Mitochondrial Dysfunction Mediated by NRF2/xCT/GPX4 Axis

**DOI:** 10.7150/ijbs.96748

**Published:** 2024-09-23

**Authors:** Junyan Wang, Haowen Zhuang, Xiaocui Yang, Zhijiang Guo, Kainan Zhou, Nanyang Liu, Yang An, Ye Chen, Zhongzheng Zhang, Mengyuan Wang, Jinhong Chen, Chun Li, Xing Chang

**Affiliations:** 1 School of Pharmaceutical Sciences, Guangzhou University of Chinese Medicine, Guangzhou, Guangdong, 510006, China.; 2 State Key Laboratory of Dampness Syndrome of Chinese Medicine, The Second Affiliated Hospital of Guangzhou University of Chinese Medicine, Guangzhou, Guangdong, 510006, China.; 3 The Second Affiliated Hospital of Liaoning University of Traditional Chinese Medicine, 110032, China.; 4 Guang'anmen Hospital, China Academy of Chinese Medical Sciences, Beijing, 100053, China.; 5 Xiyuan Hospital, China Academy of Chinese Medical Sciences, Beijing, China.; 6 Liaoning University of Traditional Chinese Medicine, Shenyang, Liaoning, 110032, China.; 7 Xianning Medical College, Hubei University of Science & Technology, Xianning, 437000, China.; 8 State Key Laboratory of Traditional Chinese Medicine Syndrome, Guangzhou University of Chinese Medicine, Guangzhou, Guangdong, 510006, China.

**Keywords:** sappanone A, Nrf-2/GPX-4/xCT, Non-small-cell carcinoma, mitochondrial, ferroptosis

## Abstract

Non-small cell lung cancer (NSCLC), a major subtype of lung cancer, encompasses squamous cell carcinoma, adenocarcinoma, and large cell carcinoma. Compared to small cell lung cancer, NSCLC cells grow and divide more slowly, and their metastasis occurs at a later stage. Currently, chemotherapy is the primary treatment for this disease. Sappanone A (SA) is a flavonoid compound extracted from the plant Caesalpinia sappan, known for its antitumor, redox-regulating, and anti-inflammatory properties. Recent studies have investigated the interaction of SA with mitochondrial pathways in regulating cell death through the Nrf-2/GPX-4/xCT axis. This study specifically explores the mechanism by which SA affects mitochondrial morphology and structure through the regulation of mitophagy and mitochondrial biogenesis in tumor cells. The study primarily utilizes second-generation transcriptomic sequencing data and molecular docking techniques to elucidate the role of SA in regulating programmed cell death in tumor cells. The omics results indicate that SA treatment significantly targets genes involved in oxidative phosphorylation, mitophagy, mitochondrial dynamics, and oxidative stress. Further findings confirmed that the Nrf-2/GPX4/xCT pathway serves as a crucial target of SA in the treatment of NSCLC. Knockdown of Nrf-2 (si-Nrf-2) and Nrf-2 overexpression (ad-Nrf-2) were shown to modulate the therapeutic efficacy of SA to varying degrees. Additionally, modifications to the GPX4/xCT genes significantly affected the regulatory effects of SA on mitochondrial autophagy, biogenesis, and energy metabolism. These regulatory mechanisms may be mediated through the caspase pathway and ferroptosis-related signaling. Molecular biology experiments have demonstrated that SA intervention further inhibits the phosphorylation of FUNDC1 at Tyr18 and downregulates TOM20 expression. SA treatment was found to reduce the expression of PGC1α, Nrf-1, and Tfam, resulting in a decrease in mitochondrial respiration and energy metabolism. Overexpression of Nrf-2 was shown to counteract the regulatory effects of SA on mitophagy and mitochondrial biogenesis. Confocal microscopy experiments further revealed that SA treatment increases mitochondrial fragmentation, subsequently inducing mitochondrial pathway-mediated programmed cell death. However, genetic modification of the Nrf-2/GPX4/xCT pathway significantly altered the regulatory effects of SA on tumor cells. In conclusion, SA has been identified as a promising therapeutic agent for NSCLC. The mitochondrial pathway-mediated apoptosis and ferroptosis may represent key mechanisms in regulating tumor cell death. Targeting the Nrf-2/GPX-4/xCT axis offers a novel therapeutic approach for maintaining mitochondrial homeostasis within the cellular microenvironment.

## Introduction

Lung cancer is among the most prevalent cancers worldwide, with 2.2 million new cases annually, representing 11.4% of all malignant tumors^1^. Annually, 1.8 million deaths are attributed to lung cancer, making up 18.0% of all cancer-related deaths. NSCLC, synonymous with "non-small cell carcinoma," is a major subtype of lung cancer, including squamous cell carcinoma, adenocarcinoma, and large cell carcinoma^2^. Compared to small cell lung cancer, NSCLC cells grow and divide at a slower rate, with metastasis occurring relatively later. NSCLC constitutes approximately 80-85% of all lung cancer cases. In the treatment of NSCLC, targeted therapy is currently one of the most commonly employed clinical approaches^3^. However, due to issues such as drug resistance and the high survivability of tumor cells, exploring novel therapeutic agents or identifying relevant target inhibitors has become a critical scientific challenge.

Multiple studies have demonstrated that mitochondrial influence on lung cancer primarily involves mitochondrial biogenesis, structural changes, and metabolic alterations^4^. As research into mitochondrial mechanisms advances, it has become evident that mitochondria play a regulatory role in inter-organelle signal transduction and in multiple pathways of programmed cell death, including pyroptosis, apoptosis, necroptosis, and ferroptosis^5^. The disruption of mitochondrial homeostasis is crucial in lung cancer development and invasion. Furthermore, mitochondrial regulation has been linked to drug resistance during clinical treatments, making it a key focus in mitochondrial pathway-based drug therapies^6^.

Mitochondria are membrane-bound organelles present in nearly all eukaryotic cells, primarily generating the energy required for tumor cell survival and activity through oxidative phosphorylation and the mitochondrial respiratory chain^7^. Recent studies have shown that mitochondria can influence lung cancer occurrence, progression, and drug resistance through various pathways^8^, ^9^. Targeting mitochondria with therapeutic agents or combining them with natural compounds has emerged as a popular research focus in lung cancer treatment^10^, ^11^. Mitochondria, ubiquitously present in human cells, are double-membraned organelles composed of an outer membrane, intermembrane space, inner membrane, and matrix.^12^, ^13^. Their primary function is to break down organic molecules like glucose and fatty acids through oxidative phosphorylation, providing energy to cells in the form of adenosine triphosphate (ATP)^14^, ^15^. Mitochondria also play roles in regulating the production and metabolism of reactive oxygen species (ROS), signal transduction, and the control of apoptosis^16^, ^17^.

Recent studies have also revealed that mitochondria are involved in various aspects of cancer biology, including oncogene activation, altered energy metabolism, oxidative stress, and mitochondrial dynamics, all of which are regulated by mitochondria^18^, ^19^. This regulation either directly triggers or indirectly promotes tumor development and invasion^20^.

Approximately 90% of cellular ATP is produced through this oxidative phosphorylation (OXPHOS) process in the mitochondria^20^. However, most cancer cells exhibit metabolic characteristics that are fundamentally different from those of normal tissues. Due to the hypoxic conditions in the tumor microenvironment, mitochondrial ATP production shifts from oxidative phosphorylation to a high rate of glycolysis, a phenomenon known as the Warburg effect^7^. This metabolic shift meets the heightened energy demands of tumor cells, providing a growth advantage and is considered a hallmark of cancer^21^. Current research has identified Nrf-2 as a key regulatory gene mediating mitochondrial energy metabolism and biogenesis^22^, ^23^.

Our findings suggest that mitochondrial dysfunction is a key factor in the regulation of mitochondrial activity in tumor cells by SA. However, the regulatory network and upstream signaling pathways or targets remain elusive. Research conducted in our laboratory has revealed that defective mitochondrial quality control contributes to heightened mitochondrial fission, reduced mitochondrial fusion, and dysfunction in receptor-dependent or receptor-independent mitophagy, ultimately resulting in a decrease in mitochondrial biogenesis^24^, ^25^. This concept is supported by evidence connecting disrupted mitochondrial homeostasis to multiple types of programmed cell death, such as apoptosis, necroptosis and ferroptosis, via mitochondrial pathways^24^, ^26^.

Mechanistically, programmed cell death is associated with abnormal mitochondrial homeostasis, driven by Nrf-2-mediated dysregulation of mitochondrial DNA levels and biogenesis^16^, ^27^. Although the precise mechanisms regulating mitochondrial DNA and biogenesis during tumor cell proliferation and differentiation remain unclear, emerging evidence suggests that the GPX4- and xCT-mediated ferroptosis pathway plays a critical role in targeting mitochondrial homeostasis^28^, ^29^. In light of this, our further experiments using SA intervention and gene modification techniques confirmed the advantages of SA in targeting tumor cell mitochondria. This study seeks to clarify the potential advantages of SA in the treatment of NSCLC.

## Methods

### Establishment of cell culture and transfection system

NCI-H1650 (human non-small cell lung cancer cells) were provided by the Cell Platform of the Experimental Center at Guangzhou University of Chinese Medicine. The cells were maintained in RPMI-1640 medium (Gibco, USA) supplemented with 10% fetal bovine serum (FBS) and 100 U/mL of penicillin. Sappanone A (catalog number: HY-113556, purity: 99.58%) was purchased from MedchemExpress (MCE, USA). To further investigate the effects and regulatory mechanisms of SA on lung cancer cells, we used adenovirus transfection to knock down and overexpress Nrf-2/xCT and GPX-4 in lung cancer cell models, followed by treatment with different concentration gradients of SA (low, medium, and high doses) 30.

### Molecular docking

The AutoDock Vina program was used for docking studies. SA was docked with Nrf-2/xCT (SLC7A11) and GPX-4 to identify potential conformations of the ligand at the binding site and further determine the population of orientations. Protein information was converted to PDBQT files, including structures of proteins with all polar residues containing hydrogen atoms. Flexible docking of the fixed protein-ligand complex was conducted. The docking site on the protein target was specified by generating a grid box centered on the native ligand binding site, utilizing the default grid spacing 31. After the docking search was completed, the best conformation was selected. PyMol was employed to analyze the interactions within the complex protein-ligand conformations, including details of hydrogen bonding and bond lengths 32.

### Second generation transcriptome

Total RNA was extracted from three experimental groups: control group (Con), SA treatment group (SA), and SA treatment + Nrf-2 overexpression group (SA + ad-Nrf-2). Each experimental group consisted of three biological replicates. Cells were lysed, and RNA was isolated according to the manufacturer's protocol (Invitrogen, USA). The quality and integrity of the RNA samples from the three replicate groups were assessed using an Agilent 2100 Bioanalyzer (Agilent Technologies, USA), ensuring that all RNA samples had an RNA integrity number (RIN) greater than 7.0. Raw sequencing data underwent quality control using FastQC, and clean reads were aligned to the reference genome (e.g., GRCh38) using HISAT2. Differential analysis between the control (Con) and SA (SA treat) groups was performed using DESeq2, identifying significant changes in gene expression profiles 33.

### Confocal detection

Following PBS washing, cells from various groups were directly labeled with MitoTracker dye, while other cells were fixed with paraformaldehyde for 30 minutes, as outlined in previous studies. The cells were then incubated overnight at 4°C with primary antibodies (LC3, TOM20, Tfam) 34. Following this, they were incubated with fluorescent secondary antibodies for 30 minutes, and DAPI was used for nuclear staining. Imaging and nuclear staining were performed using a laser confocal microscope. Image analysis was carried out using ImageJ software.

### Western blot

Cells were further lysed in RIPA buffer containing protease inhibitors (Thermo Fisher, Cat. No. 36978) and phosphatase inhibitors (Sigma-Aldrich, Cat. No. P5726) on ice for 10 minutes. The lysates were then centrifuged at 14,000 rpm for 15 minutes at 4°C. After adding LDS sample buffer (Life Technologies, Cat. No. NP0007) 35, the samples were heated at 90°C for 10 minutes. Proteins (20 μg) were separated on 10% to 15% DS-PAGE gels and transferred onto 0.2 μm nitrocellulose membranes (Carl Roth, Cat. No. 4685.1). The membranes were blocked with 5% milk for 1 hour, then incubated overnight at 4°C with primary antibodies, followed by a 1-hour incubation with secondary antibodies. After washing with TBST, bands were visualized using the Pierce ECL Plus substrate (Life Technologies, Cat. No. 32132).

### ELISA

The supernatant from the centrifuged whole-cell lysates was analyzed for caspase-3/caspase-9 activity, as well as Complex-I/III/V activity, following the manufacturer's instructions. Experimental data were analyzed using GraphPad software 36.

### RNA isolation and quantitative real-time PCR

Total RNA was isolated from cells using TRIzol reagent (Invitrogen). An aliquot of 0.2 mL chloroform was added to every 1 mL of homogenate, and the mixture was vigorously shaken, followed by incubation on ice for 15 minutes 37. The samples were transferred to fresh tubes, and an equal volume of isopropanol was added. The supernatant was discarded, and the RNA pellets were washed with 75% ethanol by gentle inversion, followed by centrifugation at 8,000 rpm for 8 minutes at 4°C 38. The pellets were air-dried for 10 minutes and dissolved in nuclease-free water. RNA (2 μg) was reverse transcribed using M-MLV reverse transcriptase (Invitrogen). The resulting cDNA was stored at -80°C until use. Quantitative real-time PCR (RT-PCR) was performed using SYBR Select Master Mix (Roche) and the ViiA 7 Real-Time PCR System (Applied Biosystems). Ct values were extrapolated to a standard curve, and data were normalized to 18S rRNA, the housekeeping gene 39.

### ATP measurement

To measure ATP levels in cultured cells, 4 × 10^6 cells were lysed on ice and centrifuged at 13,000 g for 5 minutes at 4°C to collect the supernatant. ATP levels in the supernatant were quantified using an ATP Assay Kit (Abcam, ab83355) at a wavelength of 570 nm via a colorimetric method, followed by statistical analysis 40.

### XFe24 hippocampal energy metabolism test

Mitochondrial energy metabolism in each group of cells was evaluated using an XF-24 Extracellular Flux Analyzer (Agilent 103592-100; Seahorse Bioscience, California, USA). Cells were seeded in XF24 cell culture plates (Cat. No. 100777-004; Agilent, California, USA) to achieve 95% confluence (3-4 × 10^4 cells/well). XF unbuffered media (XF DMEM medium, pH 7.4, Cat. No. 103757-100; Agilent) and XF24 sensor cartridges were calibrated in calibration buffer overnight. The cartridge was loaded with compounds for real-time ATP rate measurements 41.

### Statistical analyses

Statistical analysis was performed using Prism 10.0 software (GraphPad). Experimental data were analyzed and presented as means ± standard error of the mean (SEM). Comparisons between two groups were performed using either parametric t-tests or non-parametric Mann-Whitney tests. One-way analysis of variance (ANOVA) with Bonferroni post hoc tests was used for comparisons between two or more groups. A p-value < 0.05 was considered statistically significant.

## Results

### Nrf-2-mediated mitochondrial biogenesis and mitophagy are the primary therapeutic targets through which SA regulates tumor cell homeostasis

**Figure 1:**
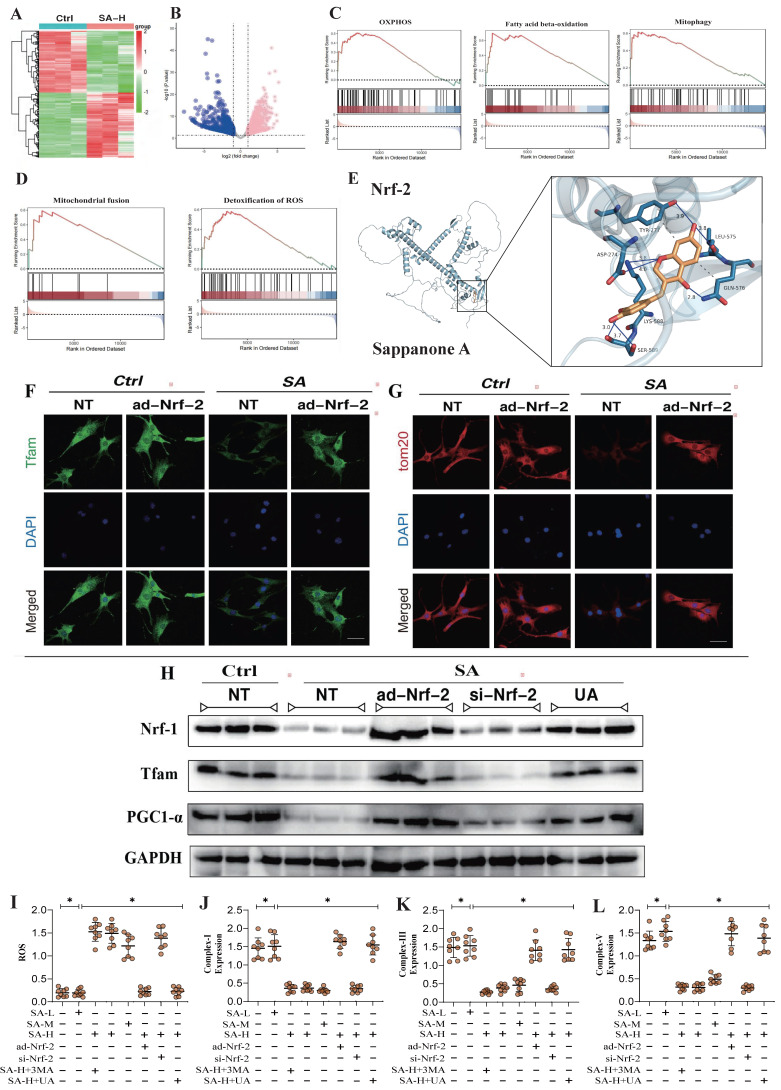
** Nrf-2-mediated mitochondrial biogenesis are the primary therapeutic targets through which SA regulates tumor cell homeostasis.** (A-B) Heatmap of gene expression in the control group and SA-H group and Volcano plot of differential genes between the control group and SA-H group; (C-D) GSEA analysis showed that compared to the control group, the tumor cells of the SA-H group mice exhibited significant activation of OXPHOS, fatty acid beta-oxidation, mitophagy, mitochondrial fusion and detoxification of ROS. (E) Molecular docking experiments showed a strong binding affinity between Nrf-2 and SA. (F-G) Laser confocal detection of mitochondrial biogenesis marker TFAM and mitophagy marker TOM20 expression level; (H) Expression of mitochondrial biosynthesis related proteins (PGC1-α/Nrf-1/Tfam); (I-L) Expression level of ROS and mitochondrial respiratory chain complex(I/III/V). Experiments were repeated at least three times and the data are shown as mean ± SEM. *p<0.05.

To investigate the SA regulates mitochondrial homeostasis in tumor cells and its upstream regulatory proteins, we employed second-generation gene sequencing to analyze the differential expression of mitochondrial-enriched genes before and after SA intervention. The results revealed that the differentially enriched genes were primarily associated with mitochondrial oxidative phosphorylation, mitophagy, mitochondrial dynamics (fusion), and oxidative stress (Figure 1A-D). These findings suggest that SA-induced disruption of mitochondrial function in tumor cells is closely related to these mechanisms. Furthermore, molecular docking experiments confirmed a strong binding affinity between Nrf-2 and SA, indicating that Nrf-2 may serve as a critical target in the therapeutic effects of SA on tumors (Figure 1E). Additionally, the experimental results showed that a high dosage of SA exhibited stronger antioxidant activity and a more significant regulation of mitochondrial respiratory chain function, whereas low and moderate doses of SA did not demonstrate the same regulatory effects (Figure 1I-L). Notably, intervention with a mitophagy activator abolished the regulatory effects of high-dose SA, while intervention with a mitophagy inhibitor did not affect the therapeutic efficacy of SA (Figure 1I-L). These findings suggest that the regulation of mitochondrial homeostasis in tumor cells by SA may be mediated through the mitophagy. Further molecular biology experiments demonstrated that SA intervention significantly suppressed the fluorescence expression of TFAM and TOM20, and reduced the protein expression of mitochondrial biogenesis-related genes, including Nrf-1, TFAM, and PGC1α (Figure 1F-H). However, overexpression of Nrf-2 and intervention with UA inhibited the regulatory effects of SA on mitochondrial biogenesis and mitophagy, whereas Nrf-2 knockdown had no impact on the therapeutic efficacy of SA (Figure 1F-H). These results suggest that the mechanism by which SA regulates mitochondrial homeostasis in lung cancer cells may involve Nrf-2-mediated mitochondrial biogenesis and mitophagy pathways.

### SA regulates programmed cell death in tumor cells through Nrf-2-mediated mitochondrial unfolded protein response

**Figure 2:**
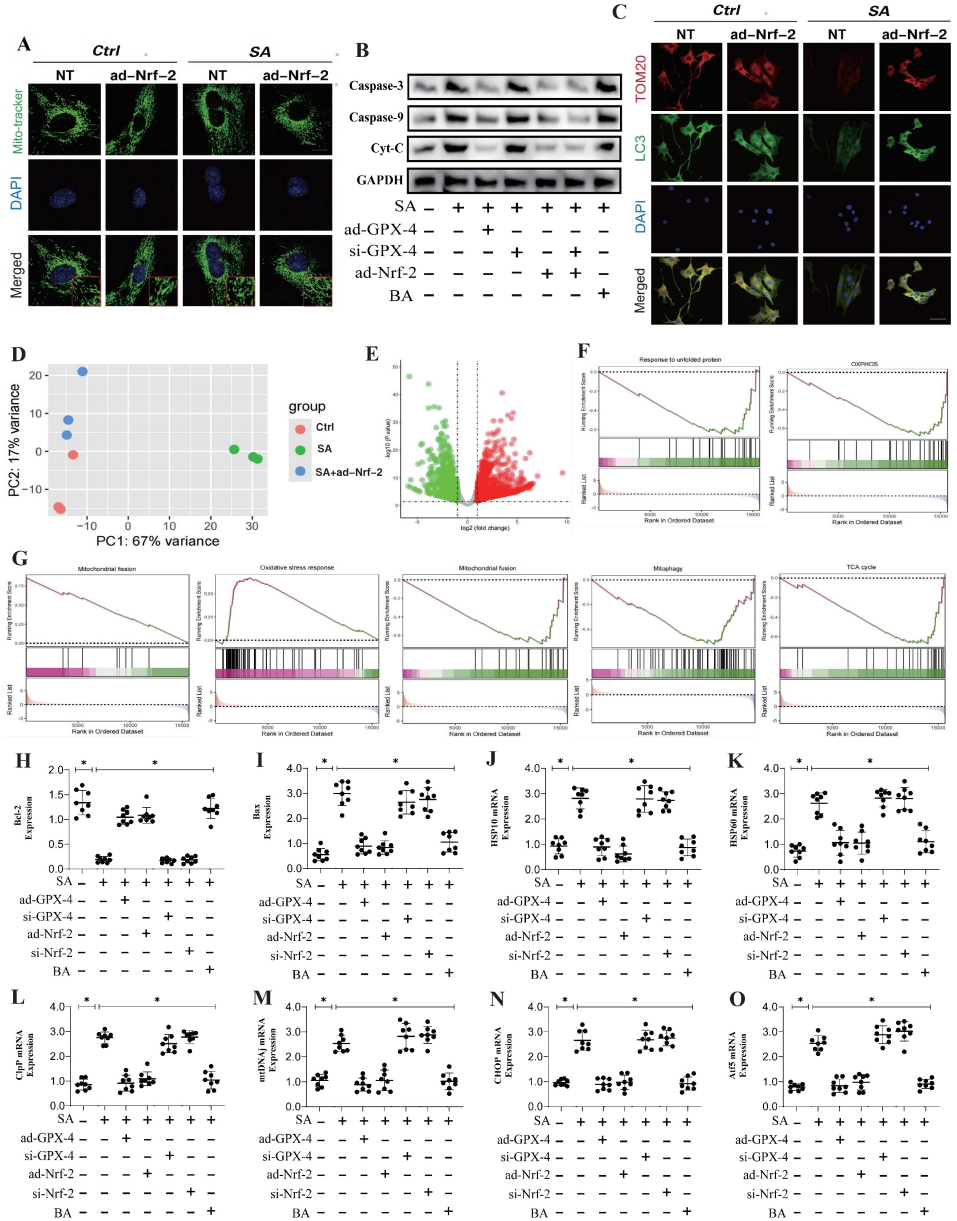
** Nrf-2-mediated Mitochondrial dynamics are important pathways for SA mediated dysregulation of cell homeostasis.** (A) Mitochondrial morphology and structural integrity testing (Mito-tracker Green); (B) Detection of expression of mitochondrial pathway-mediated apoptosis related proteins (Caspase-3/Caspase-9/Cyt-C); (C) Fluorescence co-localization detection of mitophagy related co-localization expression of LC3 and TOM20; (D) Principal component analysis between the control group, SA-H group and SA+ad-Nrf-2 group; (E) Volcano plot of differential genes between the control group; (F) GSEA analysis showed that compared to the SA group, the tumor cells of the SA+ad-Nrf-2 group exhibited significant activation of oxidative stress response, while response to unfolded protein, OXPHOS, mitochondrial fision, mitochondrial fusion, mitophagy, TCA cycle were significantly inhibited; (H-O) Apoptosis related gene expression levels (Bax/Bcl-2) and mtUPR related gene expression levels (Clpp/mtDNA/CHOP/Atf5/HSP10/HSP60); Experiments were repeated at least three times and the data are shown as mean ± SEM. *p<0.05.

To elucidate the mechanism by which SA regulates mitochondrial homeostasis through Nrf-2 and the phenotypic effects of the associated enriched genes, we conducted transcriptomic studies under Nrf-2 gene-modified conditions. The results aligned with previous findings, revealing significant differences among the NT control group, SA-treated group, and SA+ad-Nrf-2-treated group. The differentially enriched genes were primarily associated with oxidative phosphorylation, the tricarboxylic acid cycle, oxidative stress, mitophagy, and mitochondrial fusion (Figure 2D-G). Confocal imaging using Mito-Tracker showed that SA induced increased mitochondrial fission and fragmentation, along with reduced co-localization of TOM20 and LC3 (Figure 2A-C).

Further molecular biology analyses revealed abnormal expression levels of Caspase-3, Caspase-9, and Cyto-C (Figure 2A-C). Elevated mitochondrial fission levels may play a crucial role in the reduction of mitochondrial biogenesis and mitophagy, while the activation of Caspase-3, Caspase-9, and Cyto-C likely triggers mitochondrial pathway-mediated apoptosis. Notably, ad-Nrf-2/si-Nrf-2 and ad-GPX4/si-GPX4 treatments differentially influenced the regulation of Caspase-3, Caspase-9, and Cytochrome C by SA (Figure 2A-C). PCR experiments further confirmed that SA significantly upregulated the transcription of genes related to the mitochondrial unfolded protein response (UPR^mt^)(Figure 2J-O). Additionally, ad-Nrf-2/si-Nrf-2 and ad-GPX4/si-GPX4 treatments modulated the activation of the mitochondrial unfolded protein response by SA (Figure 2J-O). These findings suggest that SA may activate the mitochondrial unfolded protein response in lung cancer cells, thereby inducing mitochondrial pathway-mediated apoptosis. Analysis of Bax and Bcl-2 gene expression revealed that SA intervention activated Bax while inhibiting Bcl-2 expression (Figure 2H-I), indicating that the mitochondrial unfolded protein response induced by SA could be a key mechanism underlying mitochondrial pathway-mediated cell death.

### SA regulates mitochondrial homeostasis in tumor cells through multi-pathway mitophagy

**Figure 3:**
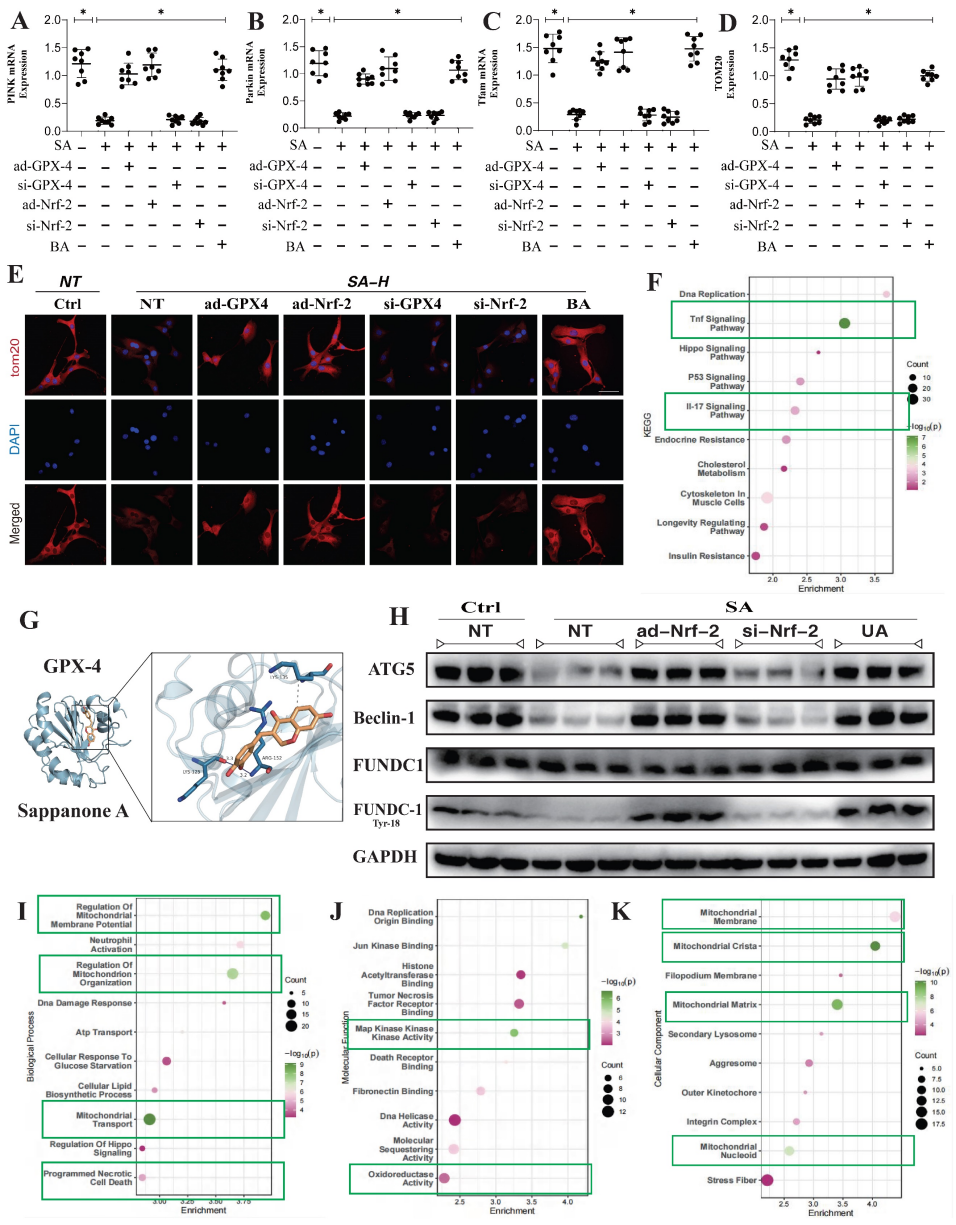
** Multi pathway mitophagy is a key mechanism by which SA regulates tumor cell homeostasis.** (A-D) Mitophagy related gene expression levels (PINK/Parkin/Tfam/TOM20); (E) Laser confocal detection mitophagy marker TOM20 expression level; (F) The KEGG analysis also demonstrates that core pathways such as Tnf signaling pathway and lL-17 signaling pathway. (G) Molecular docking experiments showed a strong binding affinity between GPX-4 and SA. (H) The expression levels of mitophagy related proteins (FUNDC-1 Tyr-18/ FUNDC-1/Beclin-1/ATG5); (I-K) GO analysis showed that the differentially expressed genes were mainly enriched in mitochondrial membrane potential, oxidoreductase activity and other aspects. Experiments were repeated at least three times and the data are shown as mean ± SEM. *P<0.05.

In addition to these findings, transcriptomic validation further confirmed that the gene differences regulated by SA through Nrf-2 may be closely associated with mitochondrial membrane potential, mitochondrial protein homeostasis, and the function of both the inner and outer mitochondrial membranes (Figure 3F,I-K). This is consistent with our previous results. However, PCR analysis related to mitophagy indicated that SA inhibited PINK/Parkin transcription and reduced TOM20 fluorescence expression, while also suppressing the expression of PGC1-α, Nrf-1, and TFAM (Figure 3A-E). Further molecular biology experiments showed that SA inhibited the phosphorylation of FUNDC1 at Tyr18 and reduced the protein expression levels of Beclin-1 and ATG5. Interventions with ad-Nrf-2/si-Nrf-2, as well as UA treatments, differentially influenced the regulation of mitophagy by SA via multiple pathways (Figure 3H). These findings suggest that the regulation of mitophagy by SA may be linked to Nrf-2-mediated mitochondrial biogenesis. Our experiments indicate that SA modulates mitophagy through both receptor-dependent autophagy, mediated by FUNDC1, and receptor-independent mitophagy, mediated by PINK/Parkin.

**Figure 4:**
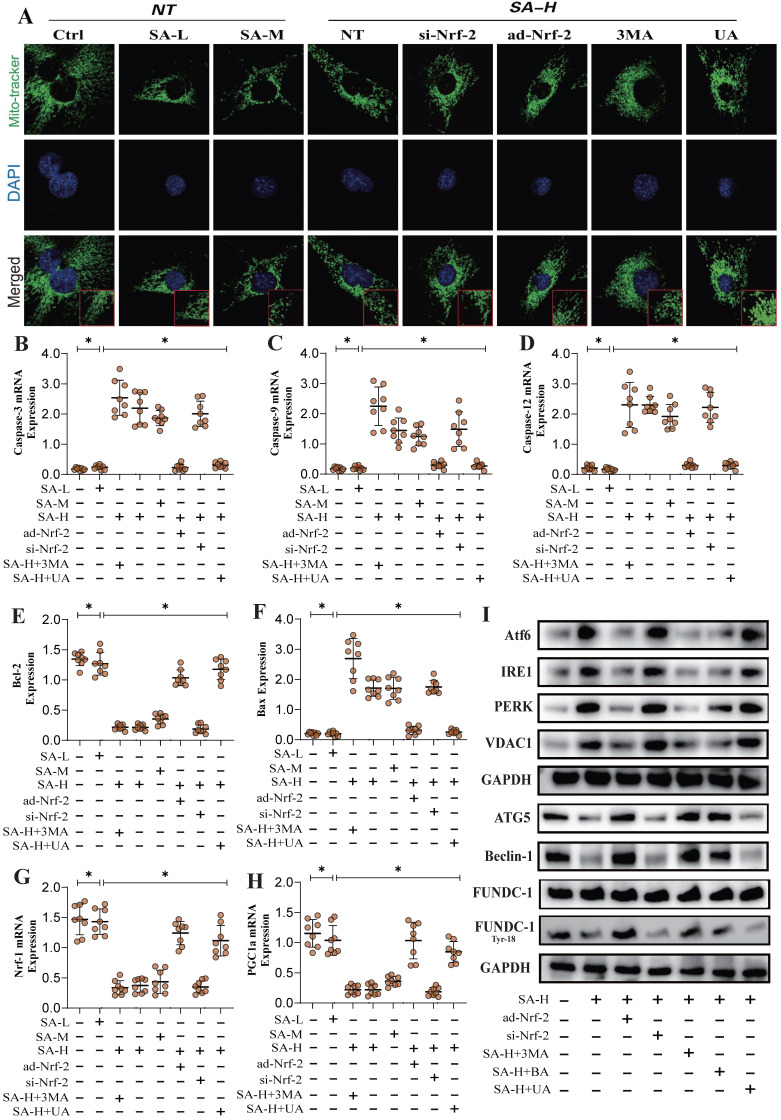
** SA regulates multiple pathways of mitochondrial autophagy through Nrf-2 and mediates mitochondrial - endoplasmic reticulum stress, activating programmed cell death.** (A) Mitochondrial morphology and structural integrity testing (Mito-tracker Green); (B) Apoptosis related gene expression levels (Bax/Bcl-2/ Caspase-3/Caspase-9/ Caspase-12) and mitochondrial biosynthesis related gene expression of levels (PGC1-α/Nrf-1); (I) Expression of mitophagy related proteins (FUNDC-1 Tyr-18/ FUNDC-1/Beclin-1/ATG5), endoplasmic reticulum stress-related genes (Atf-6/IRE1/PERK) and VDAC1. Experiments were repeated at least three times and the data are shown as mean ± SEM. *P<0.05.

To further confirm the regulatory effects of SA on mitophagy mechanisms, we examined the integrity of mitochondrial morphology and structure. The results showed that high doses of SA significantly disrupted mitochondrial morphology and structure, consistent with prior findings. Interventions with ad-Nrf-2/si-Nrf-2 and UA treatments also modulated the regulation of mitochondrial dynamics by SA to varying degrees (Figure 4A). PCR experiments related to mitochondrial pathway apoptosis further confirmed that SA disrupted the Bax/Bcl-2 balance in lung cancer cells, thereby activating mitochondrial pathway-mediated apoptosis through Caspase (Figure 4B-F). Molecular biology experiments revealed that SA treatment also activated endoplasmic reticulum stress and inhibited the phosphorylation of FUNDC1 at Tyr18, consistent with earlier findings (Figure 4I). However, low and moderate doses of SA did not significantly affect these mechanisms, while Nrf-2 gene modification and mitophagy activation negated the regulatory effects of SA (Figure 4A-I). Notably, intervention with the mitochondrial ATP synthase inhibitor BA produced the same effects as 3MA without eliminating the therapeutic efficacy of SA (Figure 4A-I). These findings suggest that SA intervention not only affects multiple pathways of mitophagy but also leads to dysfunction of the mitochondrial permeability transition pore, over-activating VDAC1 and inducing endoplasmic reticulum stress, resulting in mitochondrial energy metabolism dysfunction. This may represent a key pathway leading to mitochondrial pathway-mediated programmed cell death. However, further experiments are needed to confirm the regulatory effects of SA on mitochondrial energy metabolism pathways.

### SA regulates tumor cell mitophagy and ferroptosis through Nrf-2-mediated mitochondrial energy metabolism

**Figure 5:**
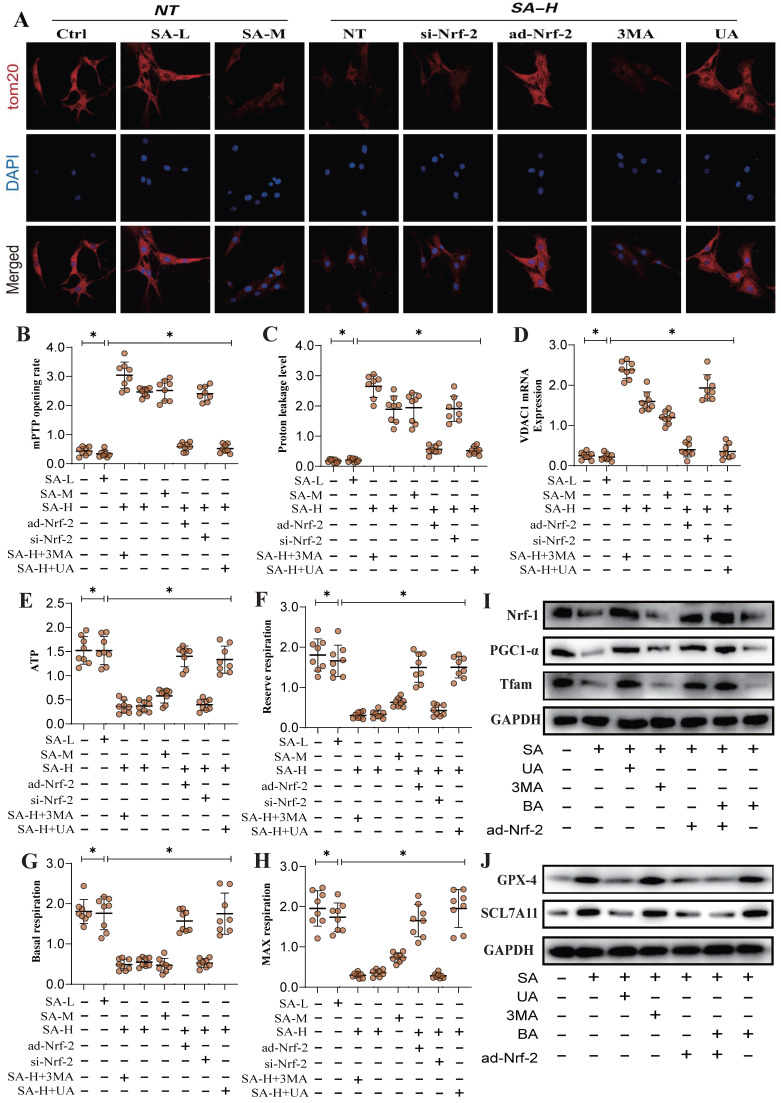
** SA regulates mitochondrial energy metabolism through Nrf-2 and mediates activation of ferroptosis.** (A) Laser confocal detection mitophagy marker TOM20 expression level; (B-H) Expression level of mPTP opening rate, proton leakage level, ATP, reserve respiration, basal respiration, max respiration and VDACI mRNA; (I-J) Expression of mitochondrial biosynthesis related proteins (PGC1-α/Nrf-1/Tfam) and ferroptosis related proteins (GPX-4/SCL7A11). Experiments were repeated at least three times and the data are shown as mean ± SEM. *P<0.05.

Mitochondrial energy metabolism is closely linked to mitophagy and programmed tumor cell death. Our results demonstrated that SA intervention may disrupt mitochondrial ATP synthase, leading to mitochondrial energy metabolism dysfunction and the inhibition of multiple mitophagy pathways. To further verify the mechanism by which SA regulates ATP synthesis and mitochondrial respiratory function through mitochondrial energy metabolism, we conducted metabolic system evaluated respiratory function in lung cancer cells. The results showed that SA reduced basal respiration, maximal respiration, reserve respiratory capacity, and ATP synthesis levels, while increasing mitochondrial proton leak (Figure 5A-H).

Additional findings suggest that SA activates ferroptosis in lung cancer cells under conditions of suppressed mitochondrial biogenesis, consistent with previous studies involving TOM20(Figure 5A-H). Nrf-2 gene modification further influenced regulation effect of SA in mitochondrial energy metabolism and biogenesis, altering its modulation of both biogenesis and ferroptosis pathways (Figure 5A-J). Dose-gradient experiments revealed that high doses of SA exhibited superior regulatory effects on mitochondrial biogenesis and ferroptosis pathways, while mitophagy activators (UA) and inhibitors (3MA) modulated the regulatory effects of SA on these mechanisms (Figure 5A-J). These findings further suggest that the regulation of mitophagy by SA through Nrf-2 may be closely associated with mitochondrial energy metabolism. Under conditions of Nrf-2-mediated biogenesis dysfunction and multi-pathway mitophagy dysfunction, mitochondrial energy metabolism is also impaired, which may be a key precursor to ferroptosis in lung cancer cells.

### SA regulates mitochondrial energy metabolism and mitochondrial biogenesis through the Nrf-2-xCT (SCL7A11) pathway

**Figure 6:**
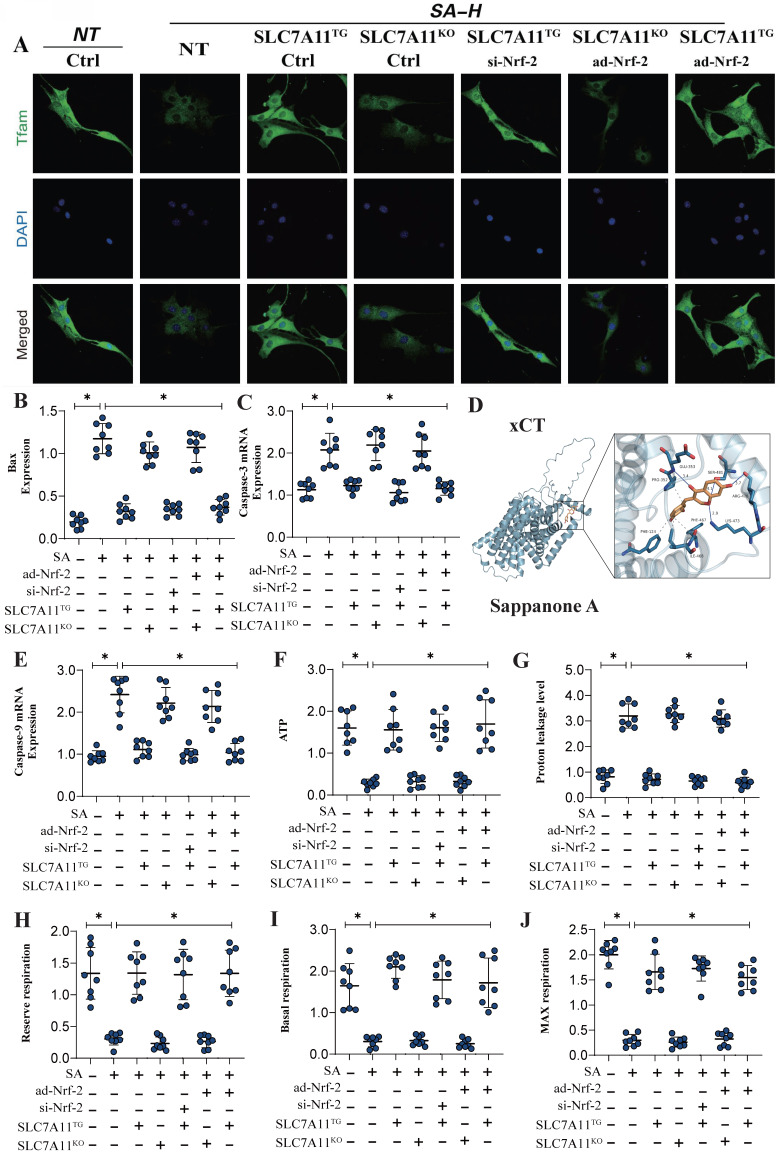
** SA regulates mitochondrial energy metabolism and mediates programmed cell death through the interaction mechanism between xCT and Nrf-2.** (A) Laser confocal detection of mitochondrial biogenesis marker TFAM; (B-E) Apoptosis related gene expression levels (Bax/Caspase-3/Caspase-9); (D) Molecular docking experiments showed a strong binding affinity between xCT and SA. (E-J) Expression level of proton leakage level, ATP, reserve respiration, basal respiration and max respiration. Experiments were repeated at least three times and the data are shown as mean ± SEM. *P<0.05.

**Figure 7:**
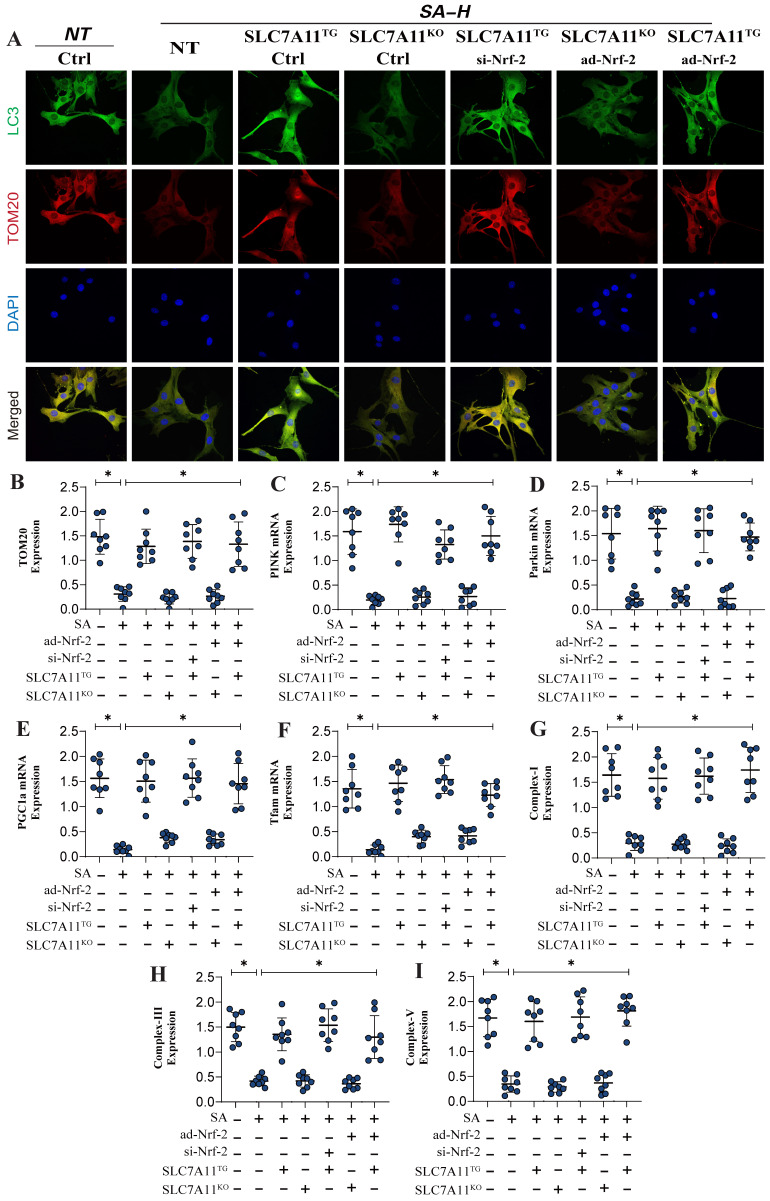
** SA regulates mitophagy and mediates mitochondrial biosynthesis dysfunction through the interaction mechanism between xCT and Nrf-2.** (A) Fluorescence co-localization detection of mitophagy related co-localization expression of LC3 and TOM20; (B-F) mitophagy related gene expression levels (TOM20/Pink1/PARKIN) and mitochondrial biosynthesis related gene expression (PGC1-α/Tfam); (G-I) Expression level of mitochondrial respiratory chain complex(I/III/V); Experiments were repeated at least three times and the data are shown as mean ± SEM. *P<0.05.

Previous experiments have confirmed that Nrf-2-mediated mitochondrial biogenesis and energy metabolism may be critical mechanisms leading to mitochondrial pathway-mediated ferroptosis. Additionally, second-generation sequencing has demonstrated that xCT (SCL7A11)-related oxidative phosphorylation and energy metabolism are key pathways through which SA regulates mitochondrial biogenesis. To further verify that the Nrf-2-xCT (SCL7A11) pathway is an important mechanism in the regulation of mitochondrial energy metabolism and programmed cell death by SA, we established an Nrf-2-xCT (SCL7A11) gene-modified lung cancer cell model. The results showed that overexpression of xCT (SCL7A11) mimicked the effects of Nrf-2 overexpression, significantly negating the regulation of mitochondrial energy metabolism (including mitochondrial respiratory function) and mitochondrial biogenesis by SA (Figure 6A,D-J). Caspase-3 and Bax assays confirmed the consistency of these findings (Figure 6B-C). However, xCT (SCL7A11) knockdown did not affect the regulatory effects of SA (Figure 6A-J). Gene modification experiments confirmed that si-Nrf-2 and ad-Nrf-2 treatments influenced the regulatory effects of SA in the context of xCT (SCL7A11) overexpression, while these treatments had no effect in the context of xCT (SCL7A11) knockdown (Figure 6A-J). These results further suggest that xCT (SCL7A11) may interact with Nrf-2, directly influencing the mitochondrial-targeted regulatory effects of SA. Further experimental results confirmed the interaction of these mechanisms, with LC3 and TOM20 fluorescence co-localization indicating that the regulation of mitophagy by SA is closely related to the xCT-Nrf-2 axis (Figure 7A-B). Transcriptional analysis of genes related to mitochondrial respiratory chain function and biogenesis was consistent with previous findings, and non-receptor-dependent mitophagy regulatory proteins PINK/Parkin also showed results consistent with earlier studies (Figure 7C-I).

Gene modification experiments further confirmed that si-Nrf-2 and ad-Nrf-2 treatments in the context of xCT (SCL7A11) overexpression influenced the regulation of PINK/Parkin-mediated mitophagy, mitochondrial biogenesis, and energy metabolism by SA. However, these treatments had no effect in the context of xCT (SCL7A11) knockdown (Figure 7C-I). In summary, our findings suggest that the xCT (SCL7A11)/Nrf-2 axis, in conjunction with GPX4, plays a critical role in the regulation of mitochondrial function by SA in lung cancer cells.

## Discussion

Our research highlights the complexity and necessity of understanding the molecular mechanisms underlying the regulation of mitochondrial damage in malignant tumor cells by SA. Current studies have yet to fully confirm the interaction between mitophagy, mitochondrial biogenesis, and ferroptosis, or the mitochondrial-targeting mechanism of SA in NSCLC. Our study utilized a series of gene-modified cell models, including Nrf-2 knockout (si-Nrf-2), Nrf-2 overexpression (ad-Nrf-2), GPX4 knockout (si-GPX4), GPX4 overexpression (ad-GPX4), xCT knockout (si-xCT), and xCT overexpression (ad-xCT). The aim was to further elucidate the pathways and mechanisms by which SA induces mitochondrial dysfunction, characterized by impaired mitophagy, increased mitochondrial fission (fragmentation), disrupted mitochondrial biogenesis, and imbalanced mitochondrial energy metabolism in tumor cells.

Our findings led to four important conclusions. First, Nrf-2 and the xCT/GPX4 axis jointly influence mitochondrial pathway-mediated programmed cell death (apoptosis and ferroptosis), identifying the regulation by SA as a critical mechanism. This provides new insights into the molecular basis of programmed cell death regulation and drug development for lung cancer. Second, under the mediation of Nrf-2 and the xCT/GPX4 axis, SA further regulates mitochondrial biogenesis and energy metabolism, impeding tumor cell proliferation and differentiation while revealing upstream inducers of programmed cell death activation. This highlights mitochondrial biogenesis and energy metabolism dysfunction as emerging phenotypes in lung cancer cell injury.

Third, SA induces significant fragmentation of mitochondrial structure, accompanied by elevated endoplasmic reticulum stress, and subsequently downregulates FUNDC1- and PINK/Parkin-mediated mitophagy. This interaction offers new mechanistic insights into mitochondrial quality control dysregulation in lung cancer cells. Fourth, Nrf-2 and the xCT/GPX4 axis interact to maintain mitochondrial homeostasis. In the absence of SA intervention, this interaction supports the regulation of mitochondrial energy metabolism in lung cancer cells. However, SA disrupts this regulatory network, suggesting that the mitochondrial-targeting mechanism of SA may be mediated through the interaction of the Nrf-2 and xCT/GPX4 axis.

Nrf-2, containing a highly conserved basic leucine zipper structure, plays a crucial role in regulating oxidative and inflammatory responses during stress and restoring metabolic homeostasis^42^, ^43^. Mitochondrial energy metabolism-driven metabolic reprogramming has been recognized as a hallmark of cancer progression. Under normal conditions, cancer cells alter their energy metabolism to meet the bioenergetic and biosynthetic demands of rapid cell proliferation, adapting to the tumor microenvironment^44^. As a key transcriptional regulator of the antioxidant stress pathway, Nrf-2 plays a critical role in metabolic reprogramming during cancer cell proliferation^45^. When growth factors stimulate normal cells, PI3K signaling activates downstream proteins that promote increased glycolytic flux and fatty acid synthesis. However, in oncogenic pathways, the PI3K pathway interacts with Nrf-2 signaling to act as a major proliferative signal, thereby regulating programmed cell death pathways in tumor cells^46^.

This further underscores the importance of Nrf-2 in regulating mitochondrial energy metabolism in tumor cells^47^. Overexpression of the transcription factor Nrf-2 in tumor cells not only promotes proliferation under various stress conditions but also induces cancer resistance^48^. The downstream target genes of Nrf-2 primarily mediate its antioxidant function, allowing tumor cells to survive in hypoxic, nutrient-deprived, and endoplasmic reticulum stress environments. Platinum-based drugs such as cisplatin and carboplatin, as well as other common chemotherapeutic agents like paclitaxel and bleomycin, induce apoptosis by raising ROS levels beyond a threshold^49^. However, sustained activation of Nrf-2 increases cancer cell resistance to ROS by upregulating antioxidant enzymes and decreasing cytotoxic sensitivity^50^. This may be one of the pathways through which Nrf-2 maintains mitochondrial homeostasis, consistent with our experimental results, which suggest that Nrf-2-mediated mitochondrial energy metabolism and biogenesis are critical mechanisms in the regulation of mitochondrial homeostasis in cancer cells by SA^51^-^53^.

In addition to the aforementioned regulatory mechanisms, mitochondrial dynamics is an important upstream regulatory mechanism of mitochondrial energy metabolism in cancer cells^54^. Mitochondrial dynamics refers to the highly dynamic nature of mitochondria maintained through cycles of fission and fusion, which govern their shape, distribution, and size^55^, ^56^. Dysregulation of this dynamic balance—divided into fusion and fission imbalances—is closely related to tumor occurrence, progression, and resistance. Regarding fusion, two members of the dynamin-related large GTPase family, MFN1, MFN2, and OPA1, regulate mitochondrial fusion^57^. Studies have confirmed that high mobility group box 1 (HMGB1) promotes the expression of phosphorylated DRP1, increasing mitochondrial fission in lung cancer cells and promoting metastasis. Furthermore, in KRAS-mutated NSCLC, DRP1 has been shown to provide energy for tumor cell growth by utilizing lactate and preventing ROS-induced oxidative clearance, thereby promoting cell proliferation^58^. This further supports the role of mitochondrial dynamics in tumor progression, aligning with our findings that SA-mediated mitochondrial fragmentation and dysfunction in mitochondrial dynamics may induce mitochondrial energy metabolism and downstream functional dysregulation, primarily affecting mitophagy activation.

Mitophagy, a subtype of the autophagy response, is crucial for regulating mitochondrial homeostasis^59^-^61^. Under conditions such as ROS stimulation, nutrient deprivation, and cellular senescence, mitochondrial membrane potential depolarizes. Depolarized mitochondria are sequestered with cellular proteins in autophagosomes, which then fuse with lysosomes for degradation, maintaining cellular homeostasis and mitochondrial adaptability^62^. In multi-pathway-regulated mitophagy, the PINK1/Parkin pathway is considered the main mechanism of mitophagy, clearing depolarized mitochondria to maintain mitochondrial function and metabolic homeostasis, preventing Warburg metabolism and excessive ROS production^60^, ^63^.

In addition to its canonical role in promoting depolarized mitochondrial autophagy, Parkin also interacts with HIF-1α to promote its degradation via ubiquitination, thereby inhibiting metastasis in breast cancer cells^64^, ^65^. HIF-1α, by inducing HIF target genes, promotes tumorigenesis, enhancing glycolysis, angiogenesis, and metastasis. Thus, loss of Parkin or PINK1 may lead to elevated HIF-1α expression, promoting tumorigenesis^66^, ^67^. Moreover, PINK1/Parkin-induced mitophagy regulates the cell cycle checkpoint by sequestering TBK1 at the centrosome, promoting spindle assembly and mitosis. Increasing mitophagy can thus inhibit cell cycle progression, suppressing the rapid proliferation of tumor cells^68^, ^69^.

Consistent with our findings, SA significantly inhibits the expression of PINK1/Parkin and ATG5, thereby suppressing mitophagy and blocking the mitophagy pathway. We hypothesize that the regulation of Warburg metabolism and mitochondrial oxidative stress by SA may be closely related to the receptor-independent mitophagy phenotype mediated by PINK1/Parkin 70.

Further research has shown that the interaction between FUNDC1 and LC3 is inhibited by SRC-mediated phosphorylation at the Y18 site within the LIR motif of FUNDC1^71^. Additional studies have confirmed that silencing FUNDC1 increases the assembly and disassembly rates of focal adhesions in prostate and glioblastoma cell lines, and increases cell motility and invasion in a DRP1-dependent manner^72^, ^73^. In contrast, overexpression of FUNDC1 limits cell migration and invasion. The enhanced migration and invasion resulting from reduced FUNDC1 expression is linked to increased liver metastasis *in vivo* but is also accompanied by decreased tumor cell proliferation. RNA-Seq analysis of 33 different tumor types from the TCGA database revealed that higher FUNDC1 levels are associated with the upregulation of genes involved in mitochondrial bioenergetics, whereas lower FUNDC1 levels are correlated with elevated ROS signaling and increased metastasis, particularly in cancer^74^, ^75^. This aligns with our findings, as our results show that FUNDC1-mediated receptor-independent mitophagy may be an important pathway through which SA regulates mitochondrial homeostasis in lung cancer cells. SA significantly inhibits activation of FUNDC1 in the TYR18 pathway, thereby inducing downstream mitochondrial dysfunction (in energy metabolism and biogenesis) and activating mitochondrial pathway-mediated apoptosis and ferroptosis 76.

Although it is still unclear whether ferroptosis involves key regulatory proteins like caspases in apoptosis, considerable evidence suggests that glutathione peroxidase 4 (GPX4) can serve as a reference marker for determining ferroptosis^77^. GPX4 plays a role in removing lipid peroxides, and its inactivation disrupts oxidative balance, allowing lipid peroxides to damage membrane structures and interact with mitochondrial regulatory proteins, triggering ferroptosis^78^-^80^. Due to its unique regulatory mechanism, GPX4 has emerged as a “star molecule,” capable of interacting with the cystine/glutamate antiporter solute carrier family 7 member 11 (SLC7A11, xCT). In tumor tissues, increased oxidative stress and enhanced nutritional metabolic demands lead to abnormal expression of GPX4 and xCT in various tumor cells, contributing to drug resistance^81^-^84^. Our results further confirm that GPX4 and xCT can interact with Nrf-2, and SA regulates this interaction mechanism, disrupting the mitochondrial internal environment and activating ferroptosis and apoptosis through the mitochondrial pathway. This provides important insights for drug research targeting multiple programmed cell death pathways in tumor cells via the mitochondrial pathway.
